# Plasminogen Activator Inhibitor 1, Cell Senescence, and Aging-Related Diseases

**DOI:** 10.3390/cells15060551

**Published:** 2026-03-19

**Authors:** Rui-Ming Liu, Mary F. Nakamya

**Affiliations:** Division of Pulmonary, Allergy, and Critical Care, Department of Medicine, School of Medicine, University of Alabama at Birmingham, 1900 University Blvd., THT 541C, Birmingham, AL 35294-0006, USA

**Keywords:** aging, PAI-1, cell senescence, aging-related diseases

## Abstract

Cellular senescence, including replicative senescence (RS) and stress-induced premature senescence (SIPS), is a state of the permanent arrest of cell growth, which can occur in proliferative cells and post-mitotic cells. Cellular senescence is believed to contribute importantly to aging and aging-related diseases. Although several hypotheses, including telomere shortening, oncogene activation, oxidative stress, DNA damage, and mitochondrial dysfunction, have been proposed, the mechanisms underlying cellular senescence in either physiological or pathological conditions remain poorly understood. Plasminogen activator inhibitor 1 (PAI-1), a physiological inhibitor of tissue type and urokinase type of plasminogen activators (tPA and uPA), has multiple functions. PAI-1 expression increases with age and in many aging-related diseases. Importantly, increased PAI-1 expression is not only a marker but also a mediator of cell senescence induced by different stimuli in vitro and in vivo. This review focuses on the recent advance in the role of PAI-1 in cell senescence during aging and in aging-related diseases as well as the potential mechanisms by which PAI-1 promotes cell senescence.

## 1. Introduction

Aging is characterized by a progressive decline in cellular and tissue functions, leading to increased susceptibility to chronic diseases. Cellular senescence, a state of irreversible cell-cycle arrest is a key mechanism driving this decline [1,2]. Cellular senescence can be triggered by telomere shortening, DNA damage, oxidative stress, oncogene activation, and environmental stressors [2,3,4]. While senescence serves as a protective mechanism in some cases, the chronic accumulation of senescent cells disrupts tissue homeostasis and contributes to pathologies in nearly every organ system [2,5,6,7,8,9,10,11,12,13]. Senescent cells are characterized by persistent DNA damage, metabolic dysfunction, and secretion of pro-inflammatory cytokines, chemokines, and growth factors, collectively known as senescence-associated secretory phenotype (SASP) [1,8,14,15]. Senescent cells also exhibit distinct phenotypic changes, including expression of senescence-associated β-galactosidase (SA-β-gal), chromatin remodeling, and extensive metabolic reprogramming [16,17,18,19,20,21]. Despite significant advancements, key questions remain regarding the heterogeneity of senescence and how its effects vary depending on cell type, tissue context, and the nature of the inducing stressor [22].

Plasminogen activator inhibitor 1 (PAI-1), also known as Serpine1, is a serine protease inhibitor and primary regulator of fibrinolysis through inhibiting the activities of tissue type of plasminogen activator (tPA) and urokinase type of plasminogen activator (uPA) [23,24]. Beyond its critical role in the maintenance of the hemostasis, PAI-1 is also involved in many biological processes, including extracellular matrix (ECM) remodeling, cell adhesion, migration, inflammation, and apoptosis, through tPA and uPA dependent- and independent mechanisms. Elevated PAI-1 is a pathological feature of many aging-related diseases, including pulmonary fibrosis [25,26,27,28], acute kidney injury (AKI) [29,30,31,32], cardiovascular diseases [33,34,35,36,37], and neurodegenerative diseases [38,39,40,41,42,43,44]. PAI-1 is also a core component of cellular senescence, serving as both a biomarker and a mediator. It is consistently up regulated in senescent cells and is a critical element of the SASP [45,46]. Importantly, inhibition of PAI-1 expression/activity genetically or pharmacologically prevents or attenuates stimuli-induced cell senescence in vitro and in vivo [28,42,45,47,48], suggesting a causative role of increased PAI-1 in cell senescence. Unlike prior reviews that examined PAI-1 within individual disease context, this work provides an integrative synthesis of the role of PAI-1 and cell senescence in aging-related diseases across several organ systems. This review also summarizes the current knowledge on the mechanisms governing PAI-1-mediating cell senescence. This framework offers a unifying perspective connecting molecular mechanisms of senescence with aging-related pathologies and highlights the translational potential of PAI-1 for aging-related diseases. No such comprehensive review has been published as our knowledge.

## 2. Cellular Senescence

Characteristics of cellular senescence: Cellular senescence is defined by three interrelated characteristics: irreversible cell-cycle arrest, metabolic reprogramming, and the SASP [5,49]. Morphologically, senescent cells adopt a flattened, enlarged shape with increased cytoplasmic granularity, reflecting cytoskeletal reorganization and accumulation of organelles, particularly enlarged lysosomes and lipid droplets [50]. A key biomarker is elevated lysosomal activity with strong SA-β-gal expression. Cytoskeletal remodeling, including the shift from filamentous (F-actin) to globular (G-actin), reduces motility and responsiveness to extracellular cues, impairing tissue repair and regenerative capacity [51,52,53]. In addition, Lamin B1 depletion disrupts nuclear architecture and promotes large-scale chromatin reorganization, including formation of SAHF. This silences proliferation-promoting genes and reinforces cell-cycle arrest [54,55]. These structural changes alter chromatin accessibility and gene expression, promote persistent DNA damage responses, and enhance pro-inflammatory signaling through the SASP [56,57]. Nuclear architectural remodeling therefore contributes to the induction and maintenance of the senescent phenotype by stabilizing senescence-associated transcriptional programs.

Irreversible cell-cycle arrest: The defining feature of senescence is irreversible proliferative arrest, first observed as the Hayflick limit [58]. This permanent growth arrest is maintained through activation of the p53/p21 and p16^INK4a^/Rb tumor suppressor pathways, which reinforce cell-cycle blockade in response to persistent DNA damage response (DDR) [54,59,60,61]. While this arrest serves as a potent tumor-suppressive barrier [62], its persistence leads to stem cell depletion, impairment of repair processes, and chronic inflammation [59,63,64]. Persistent DNA-damage foci (γH2AX, 53BP1) and nuclear remodeling characterized by Lamin B1 loss, DNA segments with chromatin alterations reinforcing senescence (DNA-SCARS), and H3K9me3-enriched senescence-associated heterochromatin foci (SAHF), cement this irreversible arrest [65,66].

Metabolic reprogramming: Cellular senescence involves profound metabolic reprogramming that sustains viability and fuels the SASP, despite of permanent growth arrest [14,67,68,69]. A defining feature is a Warburg-like shift toward aerobic glycolysis, marked by elevated glucose uptake and glycolytic enzyme expression that meet the bioenergetic and biosynthetic demands of SASP production [67,68,69,70,71,72,73,74]. Concurrently, mitochondrial dysfunction impairs oxidative phosphorylation (OXPHOS) and defines the mitochondrial dysfunction-associated senescence (MiDAS) phenotype. Defective mitochondria also overproduce reactive oxygen species (ROS) that activate p38 MAPK and NF-κB signaling, reinforcing senescence and inflammation [15,71,74,75]. Mitochondrial damage releases damage-associated molecular patterns (DAMPs), perpetuating innate immune activation and chronic inflammation [76,77]. Senescent cells exhibit altered nutrient metabolism, characterized by increased glutamine consumption, disrupted amino acid and lipid turnover, and accumulation of lipid droplets that mirror cancer-like metabolic shifts [78,79,80]. These changes sustain SASP-associated inflammation, promote nutrient competition within the tissue microenvironment, and contribute to cholesterol dysregulation and age-related metabolic diseases [81,82,83]. These metabolic and redox alterations intertwine with epigenetic regulation, as metabolites such as acetyl-CoA, α-ketoglutarate, and NAD^+^ influence chromatin remodeling, integrating metabolic flux with transcriptional control of senescence [84].

Senescence-associated secretory phenotype (SASP): SASP is another defining feature of senescence, comprising pro-inflammatory cytokines, chemokines, growth factors, matrix metalloproteinases (MMPs), and regulatory miRNA [1,16,85]. SASP arises from persistent DDR signaling via ATM-CHK2-p53 and is transcriptionally governed by NF-κB and C/EBPβ, with additional modulation through cyclic GMP-AMP synthase-stimulator of interferon genes (cGAS-STING) pathways [18,86,87,88,89,90,91]. This hypersecretory phenotype, conserved across fibroblasts, epithelial, immune, and endothelial cells, remodels the tissue microenvironment via chronic inflammation, ECM degradation, and the induction of paracrine senescence [16,17,18,87,90]. Functionally, SASP mediates both repair and pathology. In the CNS, senescent astrocytes and microglia release neurotoxic SASP factors that are potentially involved in the disruption of synaptic plasticity, blood–brain barrier (BBB) integrity, and cognitive decline [12,92,93,94,95]. In vascular tissues, senescent smooth-muscle cells secrete IL-6 and MMPs, which promote inflammation and remodeling [94]. Moreover, in skin and liver, fibroblast- and hepatocyte-derived SASP is believed to contribute to photoaging and liver fibrosis [96,97]. Accumulation of SASP-producing cells with age or in chronic stress conditions correlates with “inflammaging” that may disrupt tissue homeostasis and regeneration [98,99].

**Figure 1:**
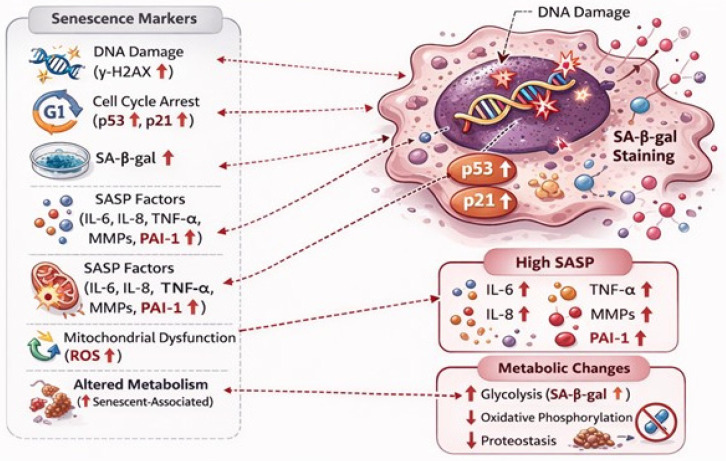
Characters of senescent cells. Senescent cells exhibit persistent DNA damage responses, illustrated by increased DNA damage (γ-H2AX) and activation of cell-cycle repressors p53, p16, and p21. A defining feature is elevated SA-β-gal activity. Senescent cells also display a robust SASP, characterized by increased secretion of pro-inflammatory and tissue-remodeling factors, including IL-6, IL-8, TNF-α, MMPs, and PAI-1. Additional hallmarks include mitochondrial dysfunction associated with increased ROS production, altered cellular metabolism characterized by enhanced glycolysis and reduced oxidative phosphorylation, and impaired proteostasis.

Thus, senescence represents a double-edged sword, transiently beneficial in tumor suppression and tissue repair, but chronically pathogenic through persistent cell-cycle arrest, metabolic dysfunction, and SASP-driven inflammation [100,101]. Figure 1 elucidates major characters of a senescent cell.

Mechanisms underlying cellular senescence: Senescence arises from interconnected molecular triggers, including telomere attrition, genotoxic stress, oncogene activation, mitochondrial dysfunction, and oxidative stress [62,102,103,104].

Telomere shortening: Telomere shortening drives replicative senescence due to progressive loss of terminal repeats and erosion of the shelterin complex (TRF1, TRF2, POT1), also called telosome), which normally protects chromosome ends [61,105,106]. Critically short telomeres activate the ATM/ATR-CHK2-p53 pathway, stabilizing p53, inducing p21^CIP1/WAF1^, and enforcing irreversible cell-cycle arrest [107,108]. Telomere dysfunction can also occur independently of length through shelterin disruption or telomeric repeat-containing RNA (TERRA) expression, activating persistent DNA damage signaling [109,110]. This process manifests across cell types. Clinically, telomere attrition is a hallmark of age-related pathologies. It drives alveolar epithelial senescence in idiopathic pulmonary fibrosis (IPF) [111,112,113], endothelial dysfunction in atherosclerosis [114,115] and exacerbates tau pathology and neuroinflammation in neurodegeneration [116,117]. Telomere dysfunction also exhibits systemic crosstalk, as shortened telomeres activate p53 to repress PGC-1α/β, reducing mitochondrial biogenesis and increasing ROS production, which reinforces senescence and SASP [118]. Thus, telomere shortening integrates genomic instability, metabolic stress, and inflammatory signaling as a central mechanism in aging.

Stress-induced premature senescence (SIPS) sections 4 and 5s a telomere-independent program triggered by genotoxic, oxidative, or inflammatory stress. DNA double-strand breaks recruit the MRE11-RAD50-NBS1 (MRN) complex, activating the ATM-CHK2 signaling pathway. This stabilizes p53 and induces p21-mediated cell-cycle arrest [119,120,121]. The resulting senescent cells are characterized by the γH2AX positive DNA damage foci and secretion of pro-inflammatory SASP factors [16,17,18]. Mitochondrial dysfunction amplifies this process through ROS production and altered NAD+/NADH ratios, creating mitochondria-derived senescence with unique SASP profiles [103,122,123,124]. Recent evidence shows that oxidative stress in nucleus pulposus cells activates the SIRT1/p53/p21 axis and SIRT1 further deacetylates p53 to delay SIPS [125]; whereas radiation-induced DDR in osteocytes drives CCL3-mediated SASP that disrupts bone homeostasis [126]. Epigenetically, p300 histone acetyltransferase promotes SIPS through chromatin loosening via H3 acetylation [127], with vascular smooth-muscle cells showing altered H3 modifications and HP1α redistribution during SIPS [128]. Experimentally, tert-butyl hydroperoxide induces mitochondrial depolarization and DNA damage in mesenchymal stem cells [129], while proton irradiation causes persistent DNA breaks across cell types [130]. Thus, SIPS integrates genotoxic stress, mitochondrial dysfunction, and epigenetic deregulation through DDR-p53/p21 signaling, with interventions targeting redox balance or histone acetylation offering potential therapeutic strategies.

Oncogene-induced senescence (OIS) is a tumor-suppressive program triggered by the aberrant activation of oncogenes like RAS, BRAF, and MYC. This activation causes excessive mitogenic signaling, replication stress, double-strand DNA breaks, and ROS accumulation, leading to a sustained DDR [62,131,132]. The DDR engages the ATM/ATR-Chk1/Chk2-p53/p21 cascade and the p16^INK4a^/Rb pathway to enforce an irreversible G1 arrest [133]. This process is reinforced by chromatin remodeling, including the formation of SAHF that represses E2F-targeted proliferation genes. OIS cells are characterized by canonical markers such as γH2AX foci, SA-β-gal activity, and a robust SASP [16,17,134,135]. The OIS state is further stabilized by PAI-1, which amplifies SASP signaling and remodels ECM [45]. The critical role of OIS as a tumor barrier is demonstrated in melanocytes, where BRAFV600E-induced senescence is abrogated by the loss of either p53 or p16^INK4a^ [131,136,137,138]. Recent findings reveal that OIS exhibits subtype-specific characteristics, including plasma membrane damage (PMD) and metabolic remodeling [139]. PMD activates Ca^2+^-dependent repair and immune-sensing pathways, enhancing SASP secretion and cytosolic DNA release, thereby reinforcing the DDR-associated senescent phenotype [133,139].

**Figure 2:**
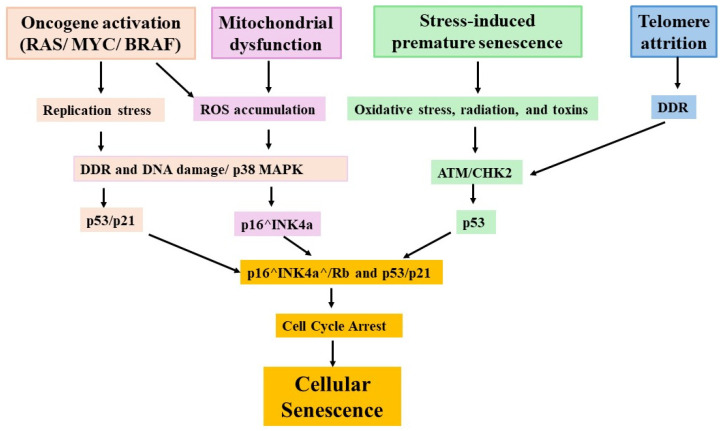
The potential mechanisms driving cellular senescence. Four major upstream stressors—oncogenic activation (RAS/MYC), mitochondrial dysfunction, genetic and oxidative stress, and telomere attrition—converge to activate DNA damage responses (DDR) and ATM, which then activate cell-cycle repression pathways p53/p16/p21 enforcing irreversible cell-cycle arrest.

Mitochondrial dysfunction is a central driver of cellular senescence, which initiates a cascade of events enforcing a permanent cell-cycle arrest [59,119,123]. Excess ROS generated by dysfunctional mitochondria damage both nuclear and mitochondrial DNA, activating the DDR-p53/p21 checkpoint pathway, while also stimulating p38 MAPK via ASK1-MKK3/6 signaling, leading to p16^INK4a^ induction [140,141,142,143]. Damaged mitochondria further release cytoplasmic mtDNA, activating cGAS-STING signaling, which amplifies SASP and promotes chronic inflammation [89,103]. Recent evidence demonstrates these mechanisms across tissues. In lens epithelial cells, La-related protein 1 (LARP1) suppressed translation of OXPHOS subunits (NDUFB8, SDHB) within stress granules, impaired ETC function, reduced ATP synthesis, and elevated ROS accumulation, leading to lens epithelial cell senescence [144]. Using knockdown and overexpression techniques, Li et al. showed, in macrophages, that NR4A1 promoted mitochondrial dysfunction and senescence through enhanced ROS production and disrupted mitochondrial potential [145]. These inter-organelle communications are part of a self-reinforcing “senescence loop” [146]. Collectively, these mechanisms are integrating with metabolic stress, genomic instability, and aberrant signaling in establishing a stable senescent state. Potential mechanisms underlying cell senescence is presented in Figure 2.

## 3. Cellular Senescence in Aging and Aging-Related Diseases

The cause of aging, an inevitable biological process that affects almost all living organisms, is still an area of significant controversy. It has long been speculated that cell senescence underlies aging and aging-related diseases involving in almost all of the organ systems [63,147,148,149,150,151,152,153,154], although the mechanisms underlying cell senescence under either physiological (normal aging) or pathological conditions remain largely undefined. Importantly, senolytic agents that specifically induce death of senescent cells, that selectively eliminate senescent cells have been shown to reduce senescent phenotypes and ameliorate pathological changes in animal studies and in clinics [153,154], further supporting a critical role of cell senescence in the development of these pathological conditions.

Cellular senescence and pulmonary disorders: Cellular senescence is believed to contribute importantly to the pathophysiology of several lung disorders, including IPF, chronic obstructive pulmonary disease (COPD), and radiation-induced pulmonary fibrosis (RIPF) [150,155,156].

Idiopathic pulmonary fibrosis is an aging-related progressive fibrotic lung disorder with unknown etiology and no effective treatment. Emerging evidence suggests that cell senescence, especially senescence of alveolar type II (ATII) cells, progenitors essential for the regeneration of alveolar epithelium [157,158,159,160], may underlie the initiation and progression of lung fibrosis. There is overwhelming evidence showing that ATII cells undergo senescence in IPF lung and in experimental lung fibrosis models [28,155,161,162,163,164,165,166,167,168,169,170,171]. Senescent ATII cells not only lose the regeneration capacity but also secrete an array of bioactive molecules, collectively called SASP. These bioactive molecules can modulate the function of secreting cells (autocrine functions) as well as adjacent cells (paracrine function), promoting surrounding cells to undergo senescence or pathological changes [168,172]. Activated fibroblasts (myofibroblasts) are the major producers of ECM and therefore play an important role in the development of lung fibrosis. Senescent fibroblasts have been identified in both primary human lung fibroblast and in bleomycin-induced pulmonary fibrosis model mice [173,174]. In both IPF tissue and bleomycin-induced rodent models, senescent fibroblasts accumulate in fibrotic foci and drive scarring through the sustained secretion of pro-fibrotic SASP factors [150,171,175,176]. A recent study from this lab showed that senescent ATII cells secrete proinflammatory cytokines/chemokines, which promote alveolar macrophage undergoing profibrogenic activation [168]. Senolytic drugs dasatinib and quercetin (D + Q) have also been shown to reduce p21-positive cells, attenuate SASP secretion, and ameliorate lung fibrosis in bleomycin-challenged mouse models [177]. Importantly, these senolytic drugs have been used in phase I clinic trial for IPF [178]. Therefore, progressive lung scarring is believed to be driven at least in part by ATII cell senescence [169,179].

Chronic obstructive pulmonary disease (COPD), which affects millions of Americans, is a disease of airways driven mainly by cigarette smoke and environmental pollutants. In bronchial and alveolar tissues from COPD patients, airway epithelial cells exhibit key senescent features, including SA-β-gal positivity, DNA-damage foci (γH2AX), p16/p21 induction, and secretion of a pro-inflammatory SASP [180,181]. This is recapitulated in vitro, where exposure to cigarette smoke extract (CSE) induces mitochondrial dysfunction, ROS accumulation, and the secretion of SASP factors such as IL-6, IL-8, and PAI-1 in primary epithelial cells [89,182,183,184]. Animal models further show that cigarette smoke-induced airway and alveolar epithelial cell senescence is exacerbated by exposure to 2.5 μm particulate matter (PM2.5), an air pollutant capable of inducing oxidative stress and inflammation [185,186,187,188]. Collectively, it is suggested that persistence of senescent epithelial, fibroblast, and endothelial cells may trigger the progression and poor repair capacity seen in COPD.

Cellular senescence in cardiovascular diseases (CVDs): Cellular senescence is a defining pathological feature in CVDs, which are a leading cause of mortality in the world, including coronary artery disease, heart failure, stroke, arrhythmia, and peripheral vascular disease [149,189,190]. Across these conditions, the accumulation of senescent cells has been detected, associated with endothelial dysfunction, fibrosis, and chronic inflammation [114,149,191,192,193,194,195]. In the vascular system, studies in ApoE^−^/^−^ and LDLR^−^/^−^ mouse models demonstrate that senescent endothelial cells exhibit telomere shortening, γH2AX DNA damage foci, reduced nitric oxide bioavailability, and increased adhesion molecules, associated with vascular inflammation and atherosclerotic plaque formation [196,197,198]. Concurrently, studies in human atheromas and animal models show that senescent vascular smooth-muscle cells (VSMCs) contribute to plaque instability, potentially through secreting pro-inflammatory and matrix-degrading enzymes [149,199,200]. In transverse aortic constriction (TAC) and isoproterenol-induced heart failure mouse models, senescent cardiomyocytes exhibit hypertrophy, mitochondrial dysfunction, and TGF-β-rich SASP secretion [201,202]. Studies in aging mouse models further show that impaired clearance of senescent cells and lost regenerative capacity of cardiac progenitor cells create a microenvironment that compromises tissue repair [191,192]. The pathological relevance is confirmed by interventional studies where senolytic treatments (Dasatinib + Quercetin or ABT263) in aged and atherosclerotic mouse models effectively clean senescent cells, restore endothelial function, and enhance myocardial performance [203,204,205].

Cellular senescence in kidney diseases: Chronic kidney disease (CKD) is a progressive disorder characterized by tubular atrophy, interstitial fibrosis, and glomerulosclerosis. In CKD, various types of senescent cells accumulate across renal compartments, including tubular epithelial cells (TECs), podocytes, mesangial cells, endothelial cells, and fibroblasts [206,207,208,209,210,211]. As demonstrated in human biopsy studies and animal models, including unilateral ureteral obstruction (UUO) and ischemia–reperfusion injury (IRI), these cells express senescence hallmarks and SA-β-gal, and secrete pro-fibrotic SASP enriched with IL-6, IL-1β, TGF-β, and PAI-1 [212,213,214]. In human diabetic nephropathy and CKD, TECs display persistent DNA damage (γH2AX foci), reduced lamin B1, and a pro-fibrotic SASP that correlates with fibrosis severity [211,215,216]. Similarly, podocytes undergo stress-induced premature senescence with upregulated p16/p21, contributing to proteinuria and glomerulosclerosis [217,218]. In db/db and STZ-induced diabetic mouse models, renal endothelial cells exhibit telomere shortening, persistent DNA damage, and reduced nitric oxide bioavailability, associated with tubulointerstitial fibrosis [206,212,215,219,220,221]. Clearance of senescent cells in INK-ATTAC transgenic mice reduced tubular atrophy by 40% and improved glomerular filtration rate by 35% following injury, whereas treatment with senolytic agents effectively eliminated senescent TECs and fibroblasts and reduced fibrosis and inflammation in UUO and IRI models [222,223,224]. These approaches establish senescence clearance as promising strategies to treat CKD [216].

Cellular senescence in neurodegenerative diseases: Alzheimer’s disease (AD) and Parkinson’s disease (PD) are two major aging-related neurodegenerative diseases [225]. Emerging evidence indicates that cellular senescence contributes importantly to the neuropathophysiology of AD and PD [42,154,226,227,228,229,230,231,232,233,234,235,236,237,238,239]. Senescent cells, including astrocytes, microglia, neurons, and endothelial cells, have been detected in the brain of AD patients [42,226,227,229,230,231,232,233,234] and in AD model mice [42,228,229,230,235]. Evidence from human and ex vivo studies using postmortem AD and PD brains reveals astrocytes with elevated p16/p21, SA-β-gal activity, and SASP expression (IL-6, IL-1β) [42,230,240]. In concert, microglia in these brains exhibit dystrophic morphology, loss of phagocytic efficiency, and elevated secretion of TNF-α and IL-1β, associated with Aβ and tau aggregation [241,242]. Studies with human AD tissue and APP/PS1 mouse models also show that oligodendrocyte progenitor cells near amyloid plaques undergo senescence; post-mitotic neurons exhibit DNA-damage foci and p21 upregulation and cerebrovascular endothelial cells display p53/p21 activation [229,243,244]. Animal models strongly support the therapeutic potential of targeting senescence as removal of senescent cells pharmacologically or genetically alleviated brain Aβ accumulation and tauopathy, two of the hallmarks of AD, and improved memory in AD model mice [228,229,230]. Similarly, in α-synuclein-overexpressing Parkinson’s disease (PD) models, dopaminergic neurons and glial cells show mitochondrial damage, increased p21 expression and SASP secretion, which are reversed by senomorphic interventions, including rapamycin and metformin [238,239]. Translationally, early-phase human trials confirm CNS penetration and tolerability of senolytic compounds, supporting the investigation of senescence as a potential therapeutic target for neurodegenerative diseases [237].

## 4. Plasminogen Activator Inhibitor 1, Aging, and Aging-Related Diseases

PAI-1 Structure and functions: PAI-1 is a single-chain glycoprotein with a molecular weight of 45–50 kDa, coded by the Serpine 1 gene on chromosome 7 (7q21.3–q22) [245]. As a member of the serine protease inhibitor (serpin) superfamily, PAI-1 comprises three β-sheets (A-C), nine α-helices (hA-hI), and a reactive center loop (RCL), which undergoes dynamic conformational shifts between active, latent, and substrate-like states to regulate its inhibitory function [246,247,248,249]. PAI-1 is synthesized and secreted as an active form, which is unstable in solution and spontaneously converts into the inactive (latent) form with a half-life about 1–2 h at 37 °C [250]. The primary function of PAI-1 is inhibition of tissue-type and urokinase-type plasminogen activators (tPA/uPA), which convert plasminogen into plasmin, a serine proteinase responsible for fibrinolysis. Therefore, the primary function of PAI-1 is maintenance of the hemostasis. In addition, PAI-1 can also block t-PA-mediated clot lysis by binding to fibrin and inactivate u-PA by internalizing u-PA and u-PA receptor (u-PAR) complex [251,252,253]. Almost all active PAI-1 in circulation binds to vitronectin, which extends its half-life by 2–10 folds. By binding to vitronectin, PAI-1 competes with u-PAR-dependent or integrin-dependent binding of cells to ECM. Therefore, PAI-1 has multiple functions and is involved in the regulation of many other biological processes, including degradation of ECM proteins, cell adhesion, migration, proliferation, apoptosis, and senescence via mechanisms independent of its antiproteolytic activity [46,254,255,256].

PAI-1 in aging and aging-related diseases: PAI-1 expression is increased in the plasma of the elderly [24,257,258,259,260,261,262], in old animals [263,264,265], in murine aging models such as Klotho-deficient (Klotho^−/−^) mice [266,267], and in many aging-related pathological conditions involving almost all organ systems [40,255,263,266,268,269]. Inhibition of PAI-1 activity or deletion of the PAI-1 gene pharmacologically or genetically ameliorates the pathophysiological changes in animal models, suggesting a critical role of increased PAI-1 in the development of these diseases.

PAI-1 and pulmonary diseases: IPF and COPD are two aging-related pulmonary diseases [168,270,271,272,273]. Studies from this lab and from others have shown that PAI-1 expression is increased in IPF and in experimental lung fibrosis models, and that increased PAI-1 may contribute to the development of lung fibrosis [28,168,170,270,274,275,276,277,278,279,280,281,282,283,284]. Our previous studies showed that inhibition of PAI-1 with a small-molecule PAI-1 inhibitor TM5257 attenuated bleomycin and TGF-β1-induced lung fibrosis in mice [279,280]. This protection was associated with enhanced fibroblast apoptosis [279,282]. Blockage of fibroblast apoptosis by PAI-1 has also been reported by others [279,282,285,286,287]. In recent studies, we further showed that specifically knocking out the PAI-1 gene whole body or in ATII cells specifically later in life ameliorated bleomycin or TGF-β1 induced ATII cell senescence, alveolar macrophage profibrogenic activation, and lung fibrosis [28,168,170,282]. Pharmacological suppression of PAI-1 with inhibitors like TM5614 in this same model also restored AT2 regenerative capacity and reduced collagen deposition [288]. Together, these studies suggest that increased PAI-1 may contribute to the development of IPF. PAI-1 is increased in the blood and sputum of COPD patients and elevated PAI-1 levels are associated with more severe airflow limitation, inflammation, and increased cardiovascular comorbidity in COPD patients [289,290,291,292]. Moreover, it has been reported that individuals with the 4G/4G genotype of the PAI-1 promoter polymorphism have a significantly higher risk of developing COPD, indicating a genetic predisposition of higher PAI-1 to COPD [36,270]. Preclinical models provide evidence supporting the role of PAI-1 in COPD. Oishi et al. reported that exposure to CSE significantly increased the inflammatory response and the PAI-1 activity, associated with destruction of lung structure and function, whereas treatment with PAI-1 inhibitor TM5441 attenuated these pathophysiological changes in a murine model of COPD [293]. Bhandary et al. also reported that PAI-1^−/−^ mice are resistant to passive CSE (PCSE)-induced lung inflammation and epithelial apoptosis in a CSE-induced COPD model [294]. Moreover, ablation of PAI-1 minimizes, whereas overexpression of PAI-1, enhanced lung injury induced by PCSE plus influenza A virus infection [294]. Together with clinical studies, it is suggested that increased PAI-1 may contribute to COPD.

PAI-1 in cardiovascular diseases (CVD): Aging is associated with increased prevalence of several cardiovascular conditions, including atherosclerosis, heart failure, and atrial fibrillation, and PAI-1 is implicated in the pathologies of these cardiovascular conditions [37,46,295,296]. Plasma level of PAI-1 is significantly elevated in Takotsubo cardiomyopathy and ischemic stroke patients [297,298] and elevated PAI-1 levels correlate with vulnerable atherosclerotic plaques [299]. In atherosclerosis, PAI-1 overexpression is associated with arterial stiffness, plaque instability, and vascular inflammation [37,46,295,296,300]. Experimental studies using mouse models further show that elevated PAI-1 expression in endothelial cells and macrophages accelerated plaque formation and neointimal thickening, whereas PAI-1 inhibition or genetic deficiency reduced lesion size and fibrosis [46,296,301]. Khoukaz et al. reported that a Western diet triggered significant upregulation of PAI-1 expression, while addition of a small molecule PAI-1 inhibitor (either PAI-039 or MDI-2268) to a Western diet dramatically reduced obesity and atherosclerosis formation, suggesting a critical role of PAI-1 in the development of atherosclerosis [301].

PAI-1 in kidney diseases: PAI-1 has been implicated in multiple aging-related renal pathologies, including CKD and diabetic kidney disease [46,247,302,303,304,305]. PAI-1 is primarily expressed in tubular epithelial cells, podocytes, and fibroblasts [302,306]. Clinical studies show that PAI-1 is elevated in plasma and urine of CKD patients and correlates with disease severity [303,304]. Chen et al. showed that high glucose and TGF-β1 induce Serpine1 expression in tubular epithelial cells, promoting tubular senescence, fibronectin accumulation, and epithelial degeneration [303]. Cohen et al. also reported that glomerular PAI-1 expression predicted the outcomes in transplanted kidneys from elderly donors and that urinary PAI-1 was associated with age-related chronic kidney disease in elderly patients [304]. Preclinical studies further support the role of PAI-1 in kidney aging and diseases [303,304,307,308]. Ma et al. found that losartan treatment in aged rats reduced renal PAI-1 expression and glomerular PAI-1 staining, coinciding with regression of glomerulosclerosis, decreased collagen, and proteinuria, suggesting that increased PAI-1 may contribute to aging-associated real fibrosis [307]. Causal evidence linking PAI-1 to kidney aging and diseases comes from the studies by Cohen [304] and Chen [303]. Cohen et al. demonstrated that deletion of PAI-1 specifically in endothelial cells in mice protected glomeruli from lesion development and podocyte loss in aged mice [304]. Their in vitro studies further showed that PAI-1 blockade inhibited podocyte apoptosis induced by senescent endothelial cell media [304]. Chen et al. demonstrated pharmacological inhibition of PAI-1 reduced senescence markers (SA-β-gal, p16^INK4A, p53) in tubular cells and attenuated degenerative changes under diabetic conditions [303]. The convergence of human genetic studies, clinical biomarker analyses, and preclinical evidence suggests that increased PAI-1 contributes to kidney aging and age-related renal disease.

**Figure 3:**
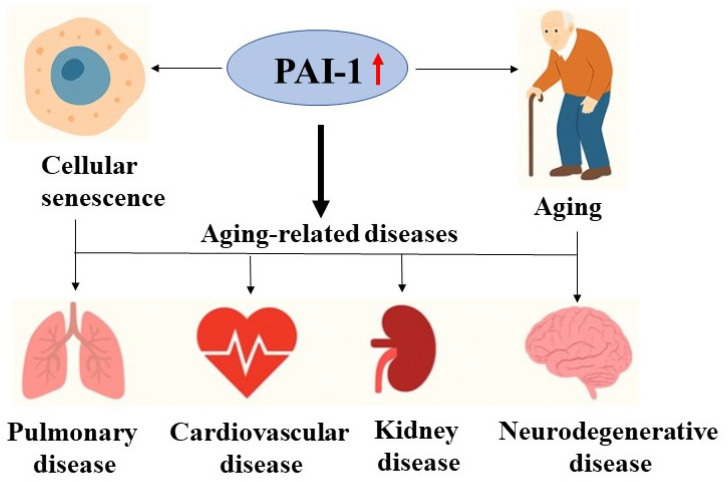
PAI-1, cell senescence and aging-related diseases. Increased PAI-1 promotes cell senescence and contributes to aging and aging-related diseases.

PAI-1 in neurodegenerative diseases: Alzheimer’s disease (LOAD), Aging is the greatest risk factor for LOAD. Although the familial Alzheimer’s disease (FAD), which accounts for less than 5% of all AD cases, is known to be caused by mutations in amyloid precursor protein (APP) and presenilin1/presenilin2 genes, the etiology for LOAD, which accounts for >95% of AD cases, remains unclear. It has been reported that PAI-1 expression is increased in AD patients [40,41,42,43,309,310,311,312] and AD model mice [40,42,43,228,235,313,314,315,316]. Mendelian randomization analyses in large human cohorts confirm that higher plasma PAI-1 levels contribute to accelerated epigenetic aging and increased Alzheimer’s susceptibility [317]. Clinical studies further show that elevated serum PAI-1 correlates with cognitive decline in aging and AD, and that exenatide therapy reduces circulating PAI-1 and improves cognition [312,318]. Using animal models, studies from different laboratories, including ours, have shown that inhibition of PAI-1 activity with small molecule PAI-1 inhibitors or knocking out the PAI-1 gene genetically reduces brain Aβ accumulation, a pathological feature of AD, and improves the memory in FAD model mice [40,43,315,316,319,320], suggesting a critical role of PAI-1 in FAD neuropathophysiology. Nonetheless, although studies have shown a critical role of PAI-1 in promoting AD pathophysiology in FAD model mice, how increased PAI-1 contributes to the neuropathophysiology of LOAD, which has clearly different etiology from FAD, is unclear. Parkinson’s disease (PD) is the second most common neurodegenerative disorder in the elderly. The disease originates from the loss of dopamine-producing neurons in the substantia nigra in the brain, resulting in unregulated activity of the basal ganglia. One of pathological features of PD is alpha-synuclein (α-syn) aggregates found in the substantia nigra region, forming Lewy bodies. Elevated serum and CSF PAI-1 levels have been reported in Parkinson’s disease patients with Lewy bodies [321,322,323,324]. Pan et al. reported that plasma PAI-1 levels were significantly increased in PD patients, compared with healthy individuals [321]. They also found that deep brain stimulation (DBS) significantly improved cognitive competence and reduced plasma PAI-1 levels in PD patients, and that plasma PAI-1 levels were negatively associated with cognitive function in PD patients [321]. Zimmermann et al. also reported that the cerebrospinal fluid levels of PAI-1 are increased in participants with Lewy body diseases, including PD, and suggest that the quantification of PAI-1 in CSF could potentially serve as a surrogate marker of aging processes [324]. Genetic studies in human populations further demonstrate that the PAI-1 gene rs2227631 and rs1799889 polymorphisms were significantly associated with PD susceptibility in the Chinese Han population, suggesting that PAI-1 has the potential to become a new therapeutic target and diagnostic marker [322]. Potential role of increased PAI-1 in driving cellular senescence, aging, and associated age-related diseases is illustrated in Figure 3.

## 5. PAI-1 and Cell Senescence

PAI-1 has emerged as a pivotal molecule in the biology of aging, not only as a biomarker but also as an active driver of cellular senescence. Increased expression of PAI-1 contributes significantly to senescence across diverse cell populations, including endothelial cells, vascular smooth-muscle cells, fibroblasts, adipocytes, epithelial cells, macrophages, and platelets, and to the aging phenotypes [25,46,170,256,304]. PAI-1 promotes cell senescence through multiple mechanisms, including reinforcing p53/p21 and p16/Rb cell-cycle checkpoints, affecting genomic stability, and amplifying the SASP signaling [28,42,45,46,47,168].

PAI-1 as a biomarker of cellular senescence: PAI-1 expression is increased under diverse stress conditions, including DNA damage and oxidative stress [325,326,327,328,329,330], two important stimuli of cell senescence. Increased expression of PAI-1 has been used as a marker of senescence for many types of cells that undergo replicative or stress-induced premature senescence, in vitro and in vivo [1,28,42,45,150,168,331,332,333,334]. Foundational in vitro studies demonstrated that human diploid fibroblast cell lines (WI-38, IMR-90) and vascular endothelial cells (HUVECs) exhibited sustained PAI-1 elevation following replicative exhaustion and telomere erosion [45,335,336]. Increased PAI-1 expression has also been demonstrated, in association with increased SA-β-gal activity, p16^INK4a^/p21^CIP1^ expression, and G1/S arrest, in many other types of cells under stress conditions, including cardiovascular and glomerular endothelial cells [304,337,338,339], uroepithelial and alveolar epithelial cells [28,168,340,341,342], and astrocytes [42]. PAI-1 is increased in the aging model mice; Klotho-deficient (*kl/kl*) mice, [267] and increased PAI-1 is used as a marker of cell senescence in various disease models in vivo [304,337,343].

PAI-1 as a mediator of cellular senescence: Numbers of in vitro and in vivo studies have shown that PAI-1 is not merely a marker but also a mediator of cellular senescence. Ghosh et al. reported that a small molecule PAI-1 inhibitor TM5441 effectively prevents doxorubicin-induced senescence in cultured cardiomyocytes, fibroblasts, and endothelial cells [344]. The same groups of the investigators also showed that treatment with TM5441 prevented L-NAME-induced p16(Ink4a) expression and telomere shortness, associated with an attenuation of periaortic fibrosis and hypertension in mice [343]. Most interestingly, they showed that PAI-1 expression is increased in aging model, Klotho-deficient (*kl/kl*) mice [267]; genetically deleting the PAI-1 gene retards the development of cell senescence, deterioration of organ structure and functions, and prolongs the lifespan of these mice [267]. This protective effect of PAI-1 inhibition against senescence-associated pathology is also well documented in bleomycin-induced murine models of pulmonary fibrosis, where PAI-1 deletion or inhibition protects against bleomycin-induced epithelial senescence and lung fibrotic remodeling [28,276,345]. Astrocyte senescence is evident in the brain of AD patients. Studies from this lab showed that overexpression of PAI-1 alone, intracellularly or extracellularly, induced senescence, whereas inhibition or silencing PAI-1 attenuated H_2_O_2_-induced senescence, in primary mouse and human astrocytes [42]. Together, the data suggest that increased PAI-1 promotes cell senescence.

Potential mechanisms underlying PAI-1 promotion of cell senescence: PAI-1 is a central driver of cellular senescence, contributing critically to the development and persistence of the senescent phenotype. It operates through multiple interconnected pathways, including cell-cycle repression, genomic instability, SASP amplification, and metabolic stress leading to an irreversible growth arrest [45,46,338].

Activation of cell-cycle repression pathways: p53 is a master cell-cycle regulator. PAI-1 is a transcriptional target of p53 [45,346,347] and also a critical regulator of p53 [28,42,46,168,170,343,344,348], creating a positive feedback loop that amplifies signaling to establish an irreversible G_1_ arrest. Studies, including ours, have shown that PAI-1 regulates the expression of multiple cell-cycle repressors, including p53, p16, and p21, in different types of cells. Boe et al. reported that treatment with a small molecule PAI-1 inhibitor TM5441 prevented N(omega)-nitro-l-arginine methyl ester (L-NAME)-induced p16(Ink4a) expression and telomere shortening in vascular cells, which was associated with an attenuation of hypertension and cardiac hypertrophy in mice [343]. The same group of investigators further reported that doxorubicin induces premature cell senescence in three cell types, endothelial cells, fibroblasts, and cardiomyocytes. Inhibition of PAI-1 with TM5441 reduced doxorubicin-mediated increases in p21, p16Ink4a and p53, and suppressed cell senescence [344]. Gifford et al. also showed that overexpression of PAI-1 in human renal epithelial (HK-2) cells increased p21 and p53 but suppress klotho expression in a kidney injury model [348]. Studies from this lab showed that silencing PAI-1 or inhibition of PAI-1 activity reduced p53 serine 18 phosphorylation, p53 and p21 protein expression, and increased retinoblastoma protein phosphorylation (pRb) in bleomycin- or doxorubicin treated rat ATII cells, whereas specific deletion of the PAI-1 gene in ATII cells in PAI_1 CKO mice led to a suppression of p53-p21-Rb cell-cycle repression pathway and lung fibrosis induced by bleomycin in mice [28]. In primary human and mouse astrocytes, silencing PAI-1 or delete the PAI-1 gene attenuated, while overexpression of PAI-1 enhanced, H_2_O_2_-induced p53 and p21 expression as well as β-gal activity [42]. Our recent studies further show that PAI-1 binds to the proteasome components, inhibits the proteasome activity and p53 degradation in human lung epithelial A549 cells and primary mouse ATII cells, suggesting that PAI-1 induces p53 at least in part through suppressing p53 degradation in the proteasome [170]. Together, these data suggest that one of the mechanisms by which PAI-1 promotes cell senescence is through activating cell-cycle repression pathways.

Promotion of SASP and inflammatory signaling: PAI-1 is a major component of the SASP [1,150,168,331,332,333,334,349]. Emerging evidence suggests that PAI-1 not only promotes SASP secretion but also mediates SASP paracrine effects. In cigarette-smoke-exposed COPD mouse models, pharmacologic PAI-1 inhibitor significantly reduces NF-κB activation, IL-6/TNF-α expression, and epithelial senescence [293], with parallel human clinical data showing sputum PAI-1 levels correlate with oxidative stress markers and macrophage NF-κB activation in COPD patients [290]. Similar mechanisms operate in other cell contexts. We showed recently that knocking out the PAI-1 gene in primary mouse astrocytes significantly attenuates H_2_O_2_-induced p16 expression and Rb dephosphorylation as well as the secretion of IL-1 and IGFBP3 [42]. Most interestingly, we showed that the condition medium (CM) from senescent astrocytes induced by transduction of astrocytes with a secretion deficient PAI-1 (no PAI-1 was secreted to the CM) had significant reduced stimulatory effect on primary neuron apoptosis, suggesting a critical role of PAI-1 in SASP paracrine effect [42]. In human endothelial stromal cells (ESCs), Griukova showed that the conditional medium form senescent ESCs induces paracrine senescence in young counterparts [349]. They identified PAI-1 to be the most prominent protein secreted by senescent ESCs, using secretome profiling techniques [349]. Using CRISPR/Cas9 techniques they further showed that PAI-1 secreted by senescent ESCs may serve as the master-regulator of paracrine senescence progression in ESCs population [349]. PAI-1 plays a critical role in tumor progression, although the underlying mechanism is not completely understood. Zhang et al. reported that chemotherapeutic agents and irradiation induce senescence in colorectal cancer (CRC) cells in vitro and in human CRC tissues [350]. Senescent tumor cells (STCs) released an increased number of EVs enriched in Serpine1, which further promoted the progression of recipient cancer cells. Targeting Serpine1 with a specific inhibitor, tiplaxtinin, markedly attenuated the tumor-promoting effect of STCs-derived EVs [350], suggesting a critical role of EVs PAI-1 in tumor progression. Mechanistically, they showed that Serpine1 bound to p65, promoting its nuclear translocation and subsequently activating the NF-κB signaling pathway [350]. In a recent study, we reported that TGF-β1 induces senesce in primary ATII cells and rat lung type 2 cell line, L2 cells, which secreted various cytokines and chemokines, including interleukin 4 (IL-4) and 13 (IL-13); the CM from these senescent cells in turn stimulates the expression of the genes associated with a pro-fibrotic phenotype in alveolar macrophages [168]. Deletion of PAI-1 or inhibition of PAI-1 activity with a small molecule PAI-1 inhibitor blocked TGF-β1-induced senescence of ATII cells as well as the stimulatory effects of the CM from senescent ATII/L2 cells on macrophages [168].

Modification of pro-senescent gene expression. Insulin-like growth factor binding protein-3 (IGFBP-3), a major IGF-binding protein in the blood, is believed to function as a pro-senescent mediator [42,334,335,344,351]. Using secretome proteomics techniques, Elzi et al. further identified IGFBP3 as a critical component in the secretome of senescent breast cancer cells [351]. Overexpression of IGFBP3 or treatment with purified recombinant IGFBP3 protein induced senescence in MCF-7 cells [351]. Most importantly, they found that tPA proteolyzes IGFBP3 and suppresses cell senescence whereas treatment with PAI-1 protein blocks tPA’s effects [351]. Ghosh et al. reported that doxorubicin induces IGFBP3 and other cell senescence markers in endothelial cells and fibroblasts whereas PAI-1 inhibitor TM5441 attenuates doxorubicin effects [344]. In a recent study, we showed that H_2_O_2_ induces p16 and IGFBP3 expression but decreases Rb phosphorylation in primary mouse astrocytes [42]. Knock out of the PAI-1 gene significantly ameliorated H_2_O_2_ effect [42]. These data suggest that PAI-1 promotes cell senescence probably in part through inhibition of proteolytic degradation of IGFBP3.

**Figure 4:**
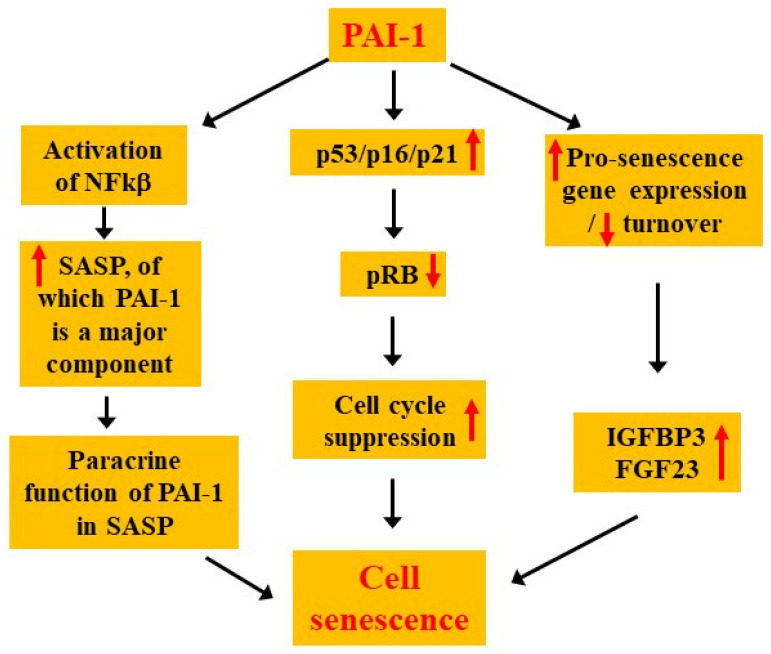
Potential mechanisms by which PAI-1 promotes cell senescence. PAI-1 promotes cell senescence through multiple mechanisms, including (1) suppressing cell-cycle through inducing or activating cell cycle repressors p53/p16/p21, (2) promotion of SASP production and amplification of SASP paracrine signals, (3) inducing or suppression turnover of pro-senescence proteins.

Fibroblast growth factor (FGF)23 is involved in the regulation of renal phosphate excretion. Serum levels of FGF23 are increased in many aging-related pathological conditions and FGF23 promotes cell senescence [352,353,354]. Importantly, studies have shown that PAI-1 is a critical regulator of FGF23 [267,354,355]. Abobulikasimu et al. showed that knocking out of the SIRT6 gene, a longevity-associated factor, in osteocytes in mice leads to increased expression of cell senescence markers, including PAI-1, p16, Il-6, and FGF23 [354]. Importantly, the SIRT6 KO phenotypes are reversed when crossing SIRT6 KO mice with PAI-1 null mice, suggesting that PAI-1 functions down stream of SIRT6, regulating FGF23 expression and cell senescence [354]. FGF23 expression is increased three-fold in PAI-1 transgenic mice whereas inhibition of PAI-1 with PAI-1 inhibitor TM5441 significantly reduced FGF23 levels in PAI-1 transgenic and Klotho-deficient mice [355]. Klotho gene functions as an aging suppressor gene. Klotho-deficient mice exhibit accelerated aging phenotypes and increased expression of FGF23 [267]. Knocking out the PAI-1 gene leads to a decrease in serum FGF23 and an extension of the life in *klotho*^−/−^ mice [267]. These data suggest that PAI-1 may mediate klotho effect on FGF23 and lifespan. These data also suggest that PAI-1 may exert its pro-senescent effect in part through inducing FGF23. Potential mechanisms by which PAI-1 promotes cell senescence is illustrated in Figure 4.

## 6. Conclusions

In conclusion, PAI-1 has emerged as a pivotal player in aging and aging-related diseases through multiple mechanisms including promoting cellular senescence. While preclinical studies demonstrate that PAI-1 inhibition mitigates senescence and improves tissue function in models of sarcopenia, pulmonary fibrosis, vascular aging, and neurodegenerative diseases clinical translation requires resolving key challenges, such as tissue-specific delivery to avoid bleeding risks from systemic fibrinolysis. Future efforts should prioritize human trials validating these inhibitors in aging-related conditions while advancing systems-level understanding of PAI-1’s interactions with thrombotic, fibrotic, and inflammatory pathways. By addressing these gaps, PAI-1 modulation could transit from mechanistic insight to a transformative strategy for extending health span and combating multimorbidity in aging populations.

## Data Availability

No need as this is a review article.
